# A Comprehensive Analysis of the Peanut SQUAMOSA Promoter Binding Protein-like Gene Family and How *AhSPL5* Enhances Salt Tolerance in Transgenic *Arabidopsis*

**DOI:** 10.3390/plants13081057

**Published:** 2024-04-09

**Authors:** Xiaohui Sun, Lili Zhang, Weihua Xu, Jianpeng Zheng, Meiling Yan, Ming Zhao, Xinyu Wang, Yan Yin

**Affiliations:** Yantai Academy of Agricultural Sciences, Yantai 265500, China; xhsun1881@163.com (X.S.); llzhang4420@126.com (L.Z.); xuweihuayt@163.com (W.X.); 13011778568@163.com (J.Z.); mlyan4373@126.com (M.Y.); mm453836637@126.com (M.Z.)

**Keywords:** peanut, SPL, *AhSPL5*, salt, gene family

## Abstract

SPL (SQUAMOSA promoter binding protein-like), as one family of plant transcription factors, plays an important function in plant growth and development and in response to environmental stresses. Despite *SPL* gene families having been identified in various plant species, the understanding of this gene family in peanuts remains insufficient. In this study, thirty-eight genes (*AhSPL1*-*AhSPL38*) were identified and classified into seven groups based on a phylogenetic analysis. In addition, a thorough analysis indicated that the *AhSPL* genes experienced segmental duplications. The analysis of the gene structure and protein motif patterns revealed similarities in the structure of exons and introns, as well as the organization of the motifs within the same group, thereby providing additional support to the conclusions drawn from the phylogenetic analysis. The analysis of the regulatory elements and RNA-seq data suggested that the *AhSPL* genes might be widely involved in peanut growth and development, as well as in response to environmental stresses. Furthermore, the expression of some *AhSPL* genes, including *AhSPL5*, *AhSPL16*, *AhSPL25*, and *AhSPL36*, were induced by drought and salt stresses. Notably, the expression of the *AhSPL* genes might potentially be regulated by regulatory factors with distinct functionalities, such as transcription factors ERF, WRKY, MYB, and Dof, and microRNAs, like ahy-miR156. Notably, the overexpression of *AhSPL5* can enhance salt tolerance in transgenic *Arabidopsis* by enhancing its ROS-scavenging capability and positively regulating the expression of stress-responsive genes. These results provide insight into the evolutionary origin of plant *SPL* genes and how they enhance plant tolerance to salt stress.

## 1. Introduction

The peanut (*Arachis hypogaea* L.) is an important global oil crop, currently grown in over 100 countries worldwide. The peanut is a type of tetraploid legume that originated approximately 9400 years ago. Nevertheless, abiotic stresses like salt, drought, cold, and heat greatly limit the quality and productivity of peanuts. With the development of bioinformatics, there have been many reports on the functional genomics of peanuts [1,2,3]. When analyzing the genetic basis of peanuts, it becomes very important to explore and analyze the functions of important genes.

Transcription factors, as important proteins in gene expression regulation, can recognize and bind to homeopathic elements to activate or inhibit downstream gene expression. This process is essential for various aspects of plant biology, including growth, hormone metabolism, and the response to stress. Currently, many transcription factors have been discovered and identified in plants, including WRKY, NAC, MYB, and so on [4]. For example, the salt-induced gene *MsWRKY33* can confer salt tolerance to alfalfa (*Medicago sativa* L.) by activating *MsERF5* and enhancing the ROS-scavenging ability [5]. The sweet potato (*Ipomoea batatas*) IbNAC3 transcription factor is involved in salt and drought compound stress responses by integrating multiple regulatory networks and ABA-dependent signaling pathways [6]. In tobacco (*Nicotiana tabacum* L.), 246 R2R3-MYB members have been identified, with the overexpression of *NtMYB102* improving salt and drought stress tolerance in transgenic tobacco [7]. SQUAMOSA promoter binding protein-like (SPL) proteins contain a highly conserved DNA binding domain, which encompasses two independent zinc finger structures, Zn-1 (Cys3His or Cys4) and Zn-2 (Cys2HisCys). SPL can recognize the GTAC element of the gene promoter to regulate the expression of downstream genes [8]. SBP was first successfully cloned from a *Antirrhinum majus* cDNA library [9]. Subsequently, *SPL* genes have been found in multiple species, including rice (*Oryza sativa*) [10], maize (*Zea mays*) [11], sweet cherry (*Prunus avium*) [12], quinoa (*Chenopodium quinoa*) [13], millet (*Setaria italica*) [8], and *Arabidopsis* [10].

It has been reported that SPL transcription factors are involved in plant growth and development, including plant embryo development [14], vegetation growth [15], flower development [16], transformations in the different developmental stages of plants [17], and maintaining plant fertility [18]. Furthermore, studies have also found that, in addition to controlling plant morphology, SPL also affects fruit quality and biomass [19,20,21,22]. Notably, SPLs are involved in plant hormone secretion and responses to biotic and abiotic stresses [23,24,25]. For example, AtSPL6 can significantly improve the resistance of *Arabidopsis* to the tobacco mosaic virus [26]. *Arabidopsis spl1-1 spl12-1* double mutant plants exhibit a sensitive phenotype under high-temperature treatment [27]. The miR156/SPL9 module enhances the freezing tolerance by activating the expression of *C-REPEAT BINDING FACTOR 2* (*CBF2*) in *Arabidopsis* [28]. TaSPL6, a transcription factor in wheat, plays a negative role in regulating the response to drought stress [29]. In rice, OsSPL10 plays an important role in the regulation of drought tolerance by directly regulating *OsNAC2* expression and ROS production [30]. The overexpression of *OsmiR529a* can enhance oxidative stress resistance by targeting the *OsSPL2* and *OsSPL14* genes [31]. In apples, salt stress tolerance is regulated by the miR156a/SPL13 module through activation of *MdWRKY100* expression [32].

The yield of peanuts is often affected by multiple abiotic stresses, especially drought and salt stresses. Increasing resistance to salt and drought is a key focus in the development of peanut varieties. Several peanut gene families have been studied, due to the peanut genome database being accessible [33,34,35]. Although the SPL family has been discovered and identified in various plants, there is still limited research on the *SPL* gene family in the peanut. For example, there are fifteen full-length cDNAs of *SPLs,* and their genomic DNA sequences have been cloned and analyzed in peanuts, revealing that the *SPL* gene is involved in the regulation of peanut growth and development [36]. In this study, we conducted a systematic analysis of the structure, location, domain, *cis*-acting elements, and expression patterns of thirty-eight peanut *SPL* genes in order to provide technical reserves for functional research on the *SPL* gene in peanuts. In addition, the overexpression of *AhSPL5* in *Arabidopsis* improved its ability to withstand high levels of salt. These findings indicate that the AhSPL members could have significant functions in peanut growth and in how peanuts react to salt and drought pressures. Moreover, these findings also provide more technical support for improving peanut yield and quality, and enhancing peanut resistance to abiotic stresses.

## 2. Results

### 2.1. Identification of the AhSPL Gene Family in Peanuts

A total of thirty-eight *AhSPL* genes were discovered through an analysis of the peanut genome database, and were subsequently designated as *AhSPL1*-*AhSPL38* based on their chromosomal positions (Supplementary Table S1). The physicochemical characteristics of the amino acid sequences suggest that the thirty-eight *AhSPL* genes encode proteins with amino acid (AA) lengths ranging from 131 (AhSPL16) to 1101 (AhSPL18), molecular weights (Mws) ranging from 15,333.8 (AhSPL16) to 122,210.08 Da (AhSPL18), and isoelectric points (pIs) ranging from 5.71 (AhSPL25) to 9.78 (AhSPL14).

### 2.2. Phylogenetic Analysis

**Figure 1:**
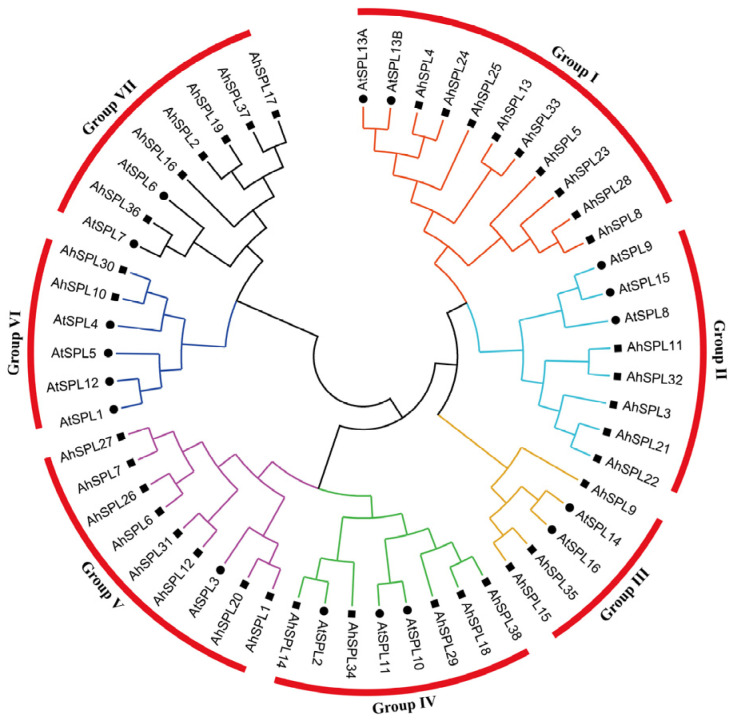
Phylogenetic analysis of AhSPL members. The phylogenetic tree was constructed using the neighbor-joining (NJ) method, aligning the SPL proteins from the peanut and *Arabidopsis*, followed by performing 1000 bootstrap replicates. Fourteen groups were created to classify the peanut SPL members and their homologs in *Arabidopsis*.

Seventeen *Arabidopsis* SPL proteins were chosen to create a neighbor-joining phylogenetic tree, along with thirty-eight peanut SPL proteins, using MEGA 6.0 software to study the evolutionary relationship of the peanut SPL family. The phylogenetic analysis showed that all of the SPL proteins were clustered into seven groups (from I to VII), and each group consisted of a minimum of one SPL protein derived from two distinct species (*Arabidopsis* and peanut) (Figure 1). The implication was that the separation between *Arabidopsis* and the peanut occurred following the divergence of the *SPL* gene family. Specifically, group I included the largest number of peanut SPL proteins (nine). Meanwhile, groups II, III, IV, V, VI, and VII consisted of five, three, five, eight, two, and six AhSPL members, respectively.

### 2.3. Conserved Motif and Gene Structure Analysis

**Figure 2:**
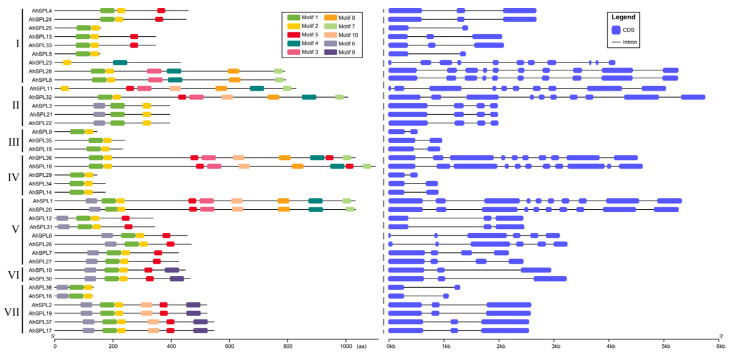
Gene structure and conserved patterns of AhSPL members.

The analysis of the gene structure of the thirty-eight *AhSPL* genes was conducted using GSDS 2.0 (Figure 2). The number of introns for the thirty-eight *AhSPL* genes ranged from one to ten. Ten and twelve *AhSPL* genes had one and two introns, respectively; seven *AhSPL* genes had nine introns; the remaining *AhSPL* genes, *AhSPL3*/*7*/*21*/*22*/*27*, *AhSPL6*/*26*, and *AhSPL18*/*23*, had three, five, and ten introns, respectively. Furthermore, the structure of the *AhSPL* genes in the same group was similar. Additionally, we analyzed the conserved motifs of the AhSPL proteins, with the MEME software (version 5.5.3) utilized for motif prediction, while the visualization of the structural protein domains was performed using Tbtools (version 1.120) (Figure 2). A total of ten motifs were identified in the AhSPL members. Among of them, motif 1 and motif 2 contained two Zn finger-like structures. Motif 2 contained nuclear localization signal (NLS) segments (Supplementary Figure S1). Notably, the members belonging to the same group had a similar motif architecture.

**Figure 3:**
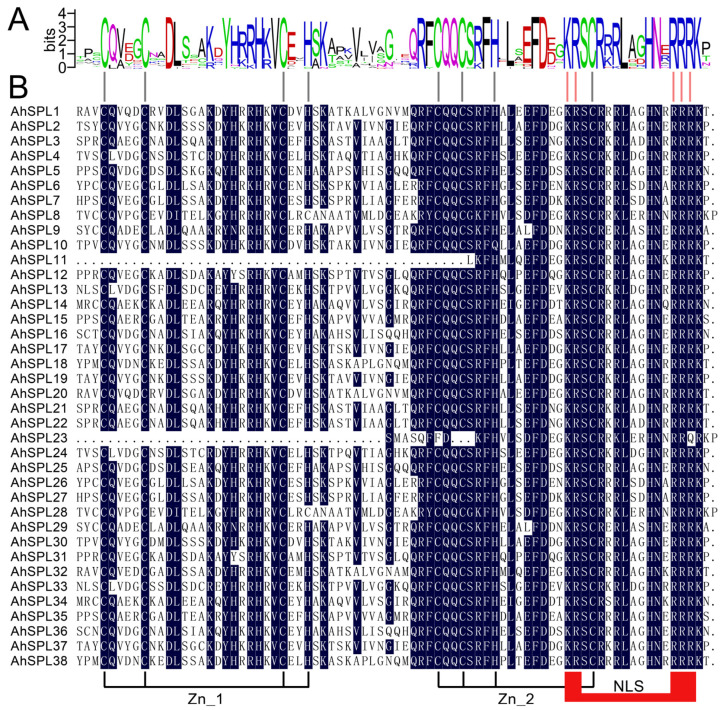
An analysis of the alignment of thirty-eight peanut SPL proteins showed both shared and distinct amino acids. (**A**) The sequence-conserved motif of the SBP domain from the AhSPL proteins. (**B**) Multiple alignments of the AhSPL proteins. Zn_1 and Zn_2 represent the zinc finger-like structures; NLS, nuclear localization signal.

The multiple alignment of all thirty-eight AhSPL proteins was conducted using DNAMAN version 6, and the SBP domain structures were subsequently presented in a detailed manner. Thirty-six AhSPL proteins possess two zinc finger-like structures (Zn_1 and Zn_2), and all the AhSPL proteins possess NLS. AhSPL11 and AhSPL23 only possess one zinc finger-like structure (Zn_2). The first zinc finger-like structure (Zn_1, Cys3His), the second zinc finger-like structure (Zn_2, Cys2HisCys), and the conserved NLS are indicated in Figure 3B. The SBP domain motif logo and protein sequence are shown in Figure 3A.

### 2.4. Chromosomal Localization, Duplication Events, and Syntenic Analysis

**Figure 4:**
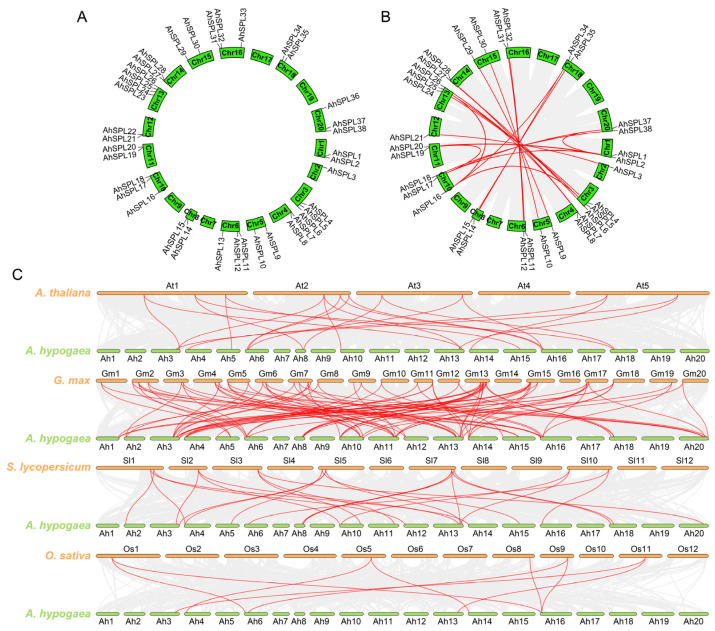
The chromosomal distribution, duplication events, and syntenic analysis of the *AhSPL* genes. (**A**) Sixteen chromosomes contained thirty-eight *AhSPL* genes that were mapped. (**B**) An MCScanX was used to analyze the segmental duplications of the twenty-four pairs of *AhSPL* genes, which are linked by the red lines. (**C**) The syntenic analysis of the *SPL* genes in the peanut and *Arabidopsis*, soybean, tomato, and rice using synteny. The collinear blocks between the peanut and the four other plant species are represented by the gray line in the background, whereas the red lines show the syntenic *SPL* gene pairs.

To improve our understanding of the specific genomic organization of the genes on the chromosomes, we constructed chromosome distribution maps for the *SPL* gene family in the peanut. The findings from our analysis of chromosome localization revealed that sixteen chromosomes in peanuts contain a total of thirty-eight *AhSPL* genes (Figure 4A). Among these chromosomes, Chr13 harbors the highest number of four *AhSPL* genes; Chr3, Chr6, Chr10, Chr16, and Chr20 contain three *AhSPL* genes; Chr1, Chr4, Chr5, Chr8, Chr11, Chr12, Chr14, Chr15, and Chr18 contain two *AhSPL* genes; while Chr2 only possesses one *AhSPL* gene. It is worth noting that no instances of tandem duplication events were observed within the peanut *SPL* gene family.

To explore the evolutionary connections among the *SPL* genes, an analysis of duplication events was conducted on the *AhSPL* genes. Among a collection of thirty-three *AhSPL* genes, twenty-four pairs of segmental duplication genes were identified (Figure 4B). The results of the duplication analysis suggest that some *AhSPL* genes may have been created through gene duplication, with segmental duplication events possibly playing a key role in the evolution of the *AhSPL* genes. It is widely accepted that a *Ka*/*Ks* ratio greater than one indicates positive selection, a *Ka*/*Ks* ratio equal to one indicates neutral selection, and a *Ka*/*Ks* ratio less than one indicates purification selection. Our findings demonstrate that all twenty-four gene pairs exhibited a *Ka*/*Ks* ratio less than 1, implying that the *AhSPL* genes underwent purification selection throughout the course of evolution (Supplementary Table S2).

Additionally, a syntenic map was constructed for the peanut, *Arabidopsis*, soybean, tomato, and rice to enhance comprehension of the evolutionary relationship among the *SPL* genes (Figure 4C). The analysis unveiled that a total of thirty-two *AhSPL* genes displayed a syntenic association with the *SPL* genes found in soybeans, followed by the tomato with twenty-one genes, *Arabidopsis* with seventeen genes, and rice with eight genes. The identified orthologous pairs between soybeans, tomato, *Arabidopsis*, and rice were found to be eighty-seven, twenty-four, twenty-one, and nine, respectively. Moreover, it was found that some *AhSPL* genes, such as *AhSPL4*, *AhSPL5*, *AhSPL12*, and *AhSPL15*, exhibit a minimum of four collinear gene pairs with soybeans. The results suggest the importance of these genes during the evolution of the peanut *SPL* gene family. Notably, *AhSPL8*, *AhSPL12*, *AhSPL28*, and *AhSPL31* show collinear relationships with the *SPL* genes from soybeans, tomato, *Arabidopsis*, and rice, indicating that these *SPL* genes may have existed before the separation of these five plant species (Supplementary Table S3).

### 2.5. Cis-Acting Elements Analysis

**Figure 5:**
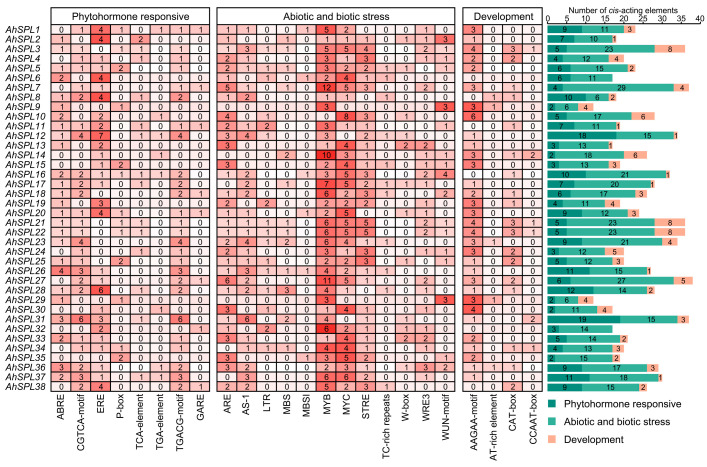
Promoter regions of *AhSPL* genes contain *cis*-acting elements.

The promoters of the *AhSPL* genes were analyzed for putative *cis*-acting elements using PlantCARE (version 1), for the upstream sequences of the thirty-eight *AhSPL* genes (2 kb upstream of the start codon). A total of twenty-four different types of these *cis*-elements were identified, including eight phytohormone responsive elements (ABRE, CGTCA-motif, ERE, P-box, TCA-element, TGA-element, TGACG-motif, and GARE), twelve abiotic and biotic stress-responsive elements (ARE, AS-1, LTR, MBS, MBSI, MYB, MYC, STRE, TC-rich repeats, W-box, WRE3, and WUN-motif), and four development-related elements (AAGAA-motif, AT-rich element, CAT-box, and CCAAT-box). Furthermore, *AhSPL27* contained the largest number of *cis*-acting elements (thirty-eight), followed by *AhSPL7* and *AhSPL31*, which contained thirty-seven elements. *AhSPL9* and *AhSPL29* contained the least number of cis-acting elements (twelve). Notably, all the *AhSPL* genes contained at least one abiotic and biotic stress-response *cis*-acting elements (Figure 5). These results indicate the significant contributions of the *AhSPL* genes to various biological processes, as well as their involvement in the response to abiotic/biotic stresses and plant hormones in peanuts.

### 2.6. Expression Pattern Analysis

**Figure 6:**
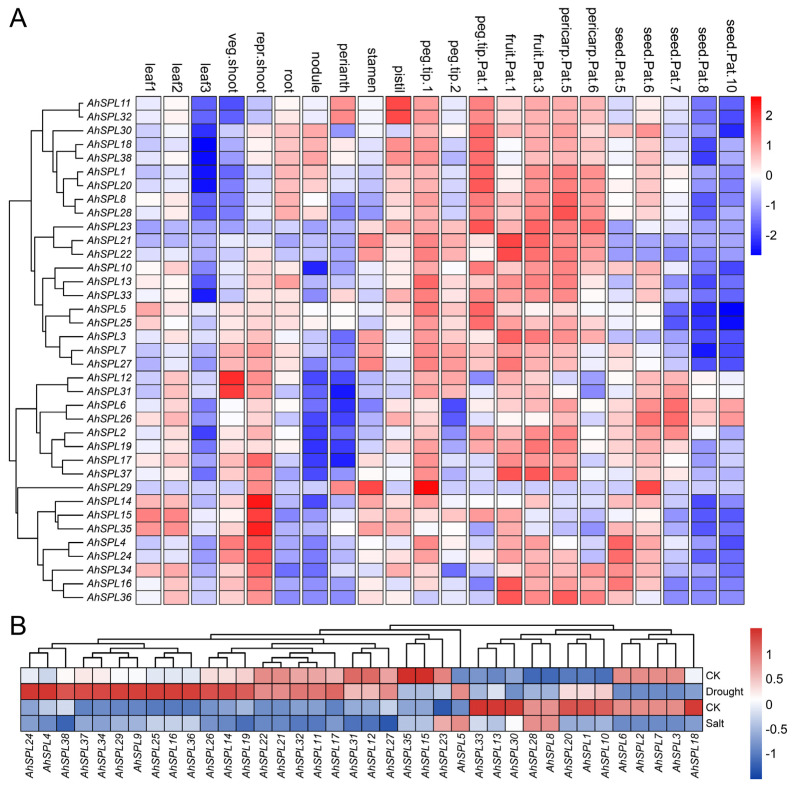
Expression analysis of *AhSPL* genes in twenty-two tissues (**A**) and in response to drought and salt stresses (**B**). CK, control untreated samples. TBtools was used to normalize and cluster the FPKM values of each *AhSPL* gene.

To comprehend the purported roles of the *AhSPL* genes, an analysis was conducted on the expression profiles of all thirty-eight identified *AhSPL* genes. This analysis utilized the presently accessible RNA-seq data for the peanut (cultivar “Tifrunner”), including twenty-two different tissues and organs: leaf, shoot, root, nodule, perianth, stamen, pistil, peg.tip, peg.tip.Pat, fruit.Pat, pericarp.Pat, and seed.Pat (Figure 6A). Eight *SPL* genes, *AhSPL4*/*14*/*15*/*16*/*24*/*34*/*35*/*36*, were highly expressed in repr.shoot; *AhSPL12* and *AhSPL31* were highly expressed in veg.shoot; thirty-two *SPL* genes, such as *AhSPL8*/*21*/*22*/*23*/*28*/*37*, were found to be highly expressed in fruit.Pat and pericarp.Pat; and *AhSPL29* was expressed at a low level in all tissues. However, *AhSPL9* was not detected in all the tested tissues (not reflected in the heat map). The varied gene expression profiles indicate that the *AhSPL* genes involved in peanut growth and development potentially possess a wide range of functions.

Furthermore, in order to gain additional understanding regarding the reaction of the *AhSPL* genes to abiotic stresses, the RNA-seq data pertaining to drought and salt stresses were acquired from the NCBI database. The cultivars J11 and Fenghua3 were used for drought and salt treatment, respectively. The results suggest that the response of the *AhSPL* genes to drought and salt treatments exhibited a noticeable disparity (Figure 6B). Under conditions of drought stress, a significant majority of the *AhSPL* genes, specifically 44.7% (seventeen out of thirty-eight), exhibited up-regulation. Conversely, the up-regulation of genes under salt stress was comparatively lower, with only 18.4% (seven out of thirty-eight) of the *AhSPL* genes displaying this response. *AhSPL1*, *AhSPL10*, *AhSPL13*, and *AhSPL19* exhibited insensitivity to salt treatment, while displaying significant induction in response to drought treatment. Significantly, *AhSPL2*, *AhSPL3*, *AhSPL6*, *AhSPL7*, *AhSPL16*, *AhSPL25*, and *AhSPL36* showed increased expression when exposed to drought and salt treatments, indicating that these seven *AhSPL* genes potentially have significant implications in the peanut’s ability to respond to salt and drought stresses.

### 2.7. Prediction of Regulatory Network

**Figure 7:**
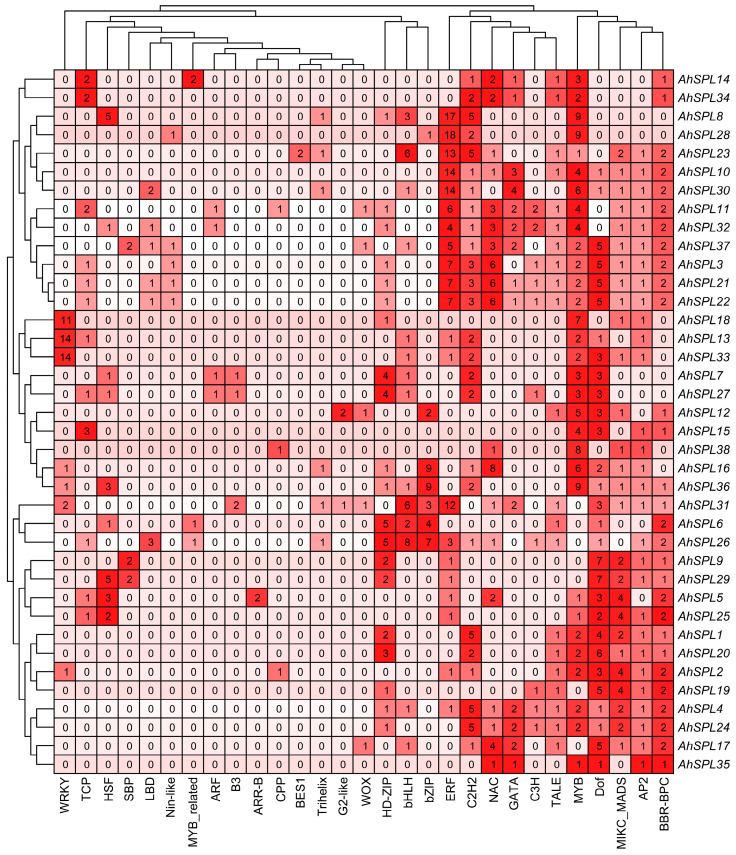
Possible transcription regulators of *AhSPL* gene expression.

We used the PlantRegMap server to predict potential regulatory interactions between the transcription factors and *AhSPL* genes. The analysis yielded a comprehensive set of twenty-nine transcription factors that were identified as potential regulators of *AhSPL* genes expression. The binding of these transcription factors to the promoters of the *AhSPL* genes were quantified and visualized through the utilization of TBtools (Figure 7). MYB, Dof, MIKC_MADS, AP2, and BBR-BPC transcription factors have the potential to regulate a significant portion of almost all of the *AhSPL* genes. The regulatory patterns of *AhSPL4*, *AhSPL21*, *AhSPL22*, and *AhSPL24* demonstrated a resemblance, suggesting that these four genes could potentially be regulated by C2H2, NAC, GATA, C3H, TALE, MYB, Dof, MIKC_MADS, AP2, and BBR-BPC transcription factors. Notably, *AhSPL8* exhibited regulation by the highest number of transcription factors (forty-one), while *AhSPL35* displayed the lowest regulation, with only six transcription factors.

**Figure 8:**
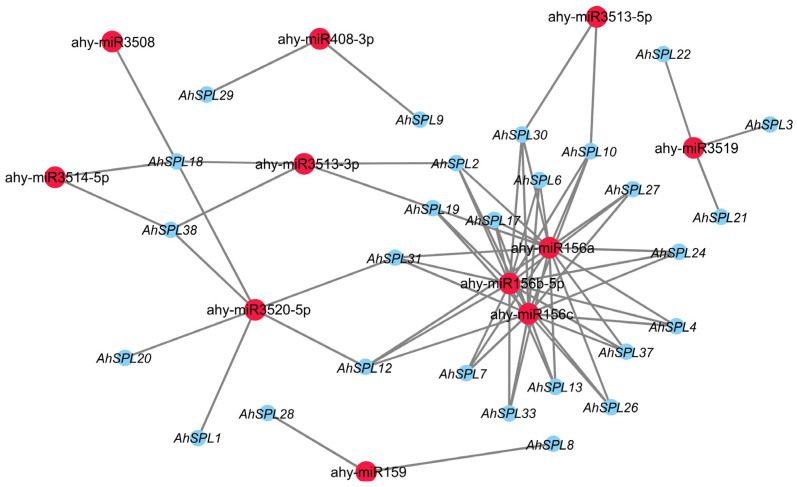
Possible microRNA regulators of *AhSPL* genes.

Previous studies have shown that microRNAs (miRNAs) are able to play an important role in plant responses to abiotic stresses by the direct regulation of the *SPL* genes [37]. Therefore, we analyzed the miRNAs that may regulate *AhSPL* genes expression by using the psRNATarget server (https://www.zhaolab.org/psRNATarget/, version 2, accessed on 26 September 2023). The results show that a total of eleven peanut miRNAs are predicted to be regulators of the *AhSPL* genes (Figure 8). Among of them, ahy-miR156a, ahy-miR156b, and ahy-miR156c regulate a maximum number of sixteen *AhSPL* genes, followed by ahy-miR3520-5p, which regulates six *AhSPL* genes. However, ahy-miR3508 regulates only the *AhSPL18* gene.

### 2.8. GO Enrichment Analysis

**Figure 9:**
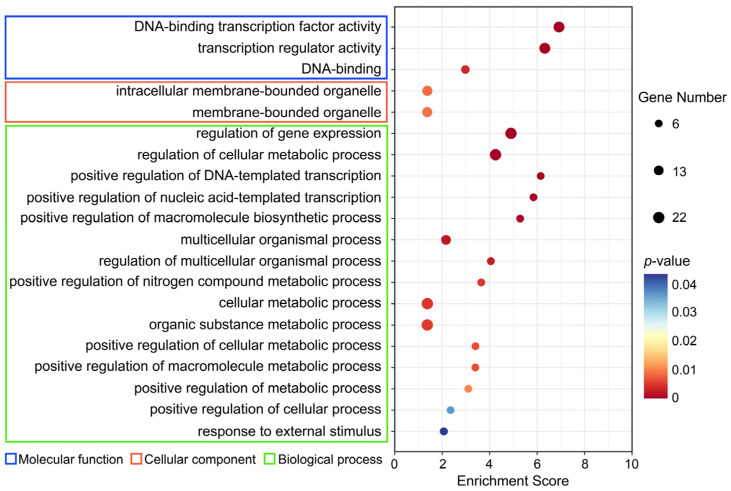
GO enrichment analysis of *AhSPL* genes.

We further explored the potential biological functions of the *AhSPL* genes by using a GO enrichment analysis. Three terms in the molecular function (MF), two terms in the cellular component (CC), and fifteen terms in the biological process (BF) were selected for presentation in Figure 9. The analysis of the MF, CC, and BP annotations suggested that the major function of these *AhSPL* genes is related to DNA-binding transcription factor activity (GO:0003700), the intracellular membrane-bounded organelle (GO:0043231), the regulation of gene expression (GO:0010468), the regulation of cellular metabolic processes (GO:0031323), the positive regulation of macromolecule biosynthetic processes (GO:0010557), the positive regulation of metabolic processes (GO:0009893), the positive regulation of cellular processes (GO:0048522), and responses to external stimuli (GO:0009605).

### 2.9. Overexpression of AhSPL5 Enhanced Salt Tolerance

**Figure 10:**
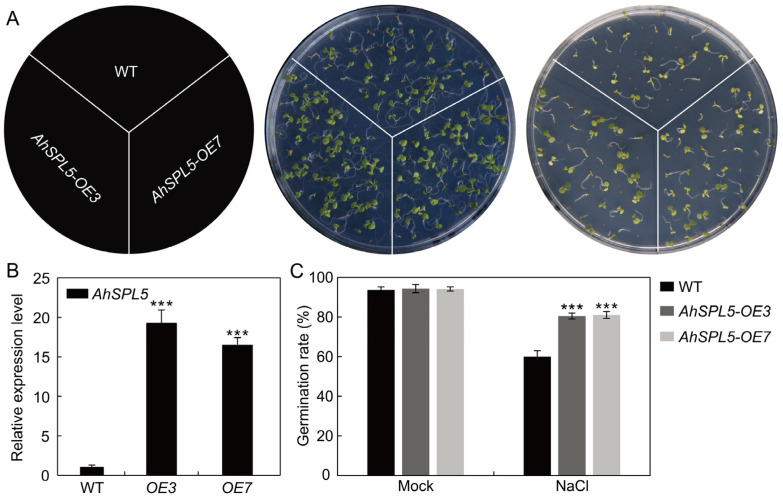
Evaluating seed germination in *AhSPL5-OE* lines and wild-type *Arabidopsis* when exposed to salt stress conditions. (**A**) Comparison of seed germination rates between *AhSPL5-OE* lines and wild-type *Arabidopsis* plants exposed to 100 mM NaCl treatments. Germination rates of seeds were documented after six days of sowing. (**B**) Expression levels of *AhSPL5* gene in *AhSPL5-OE* lines and wild-type *Arabidopsis*. (**C**) Measurement of main root lengths under control conditions and after treatment with 100 mM NaCl. WT, wild type. The data are means ± SD from three independent replications. *** *p* < 0.001 (*t*-tests).

To investigate the functions of *AhSPL5* in the context of the salt stress response, transgenic *Arabidopsis* plants were developed to overexpress *AhSPL5*. A total of ten T0 transgenic lines were confirmed through normal PCR. Subsequently, homozygous T3 transgenic lines were identified and subjected to qRT-PCR to measure the expression levels of *AhSPL5*. Two transgenic lines (*OE3* and *OE7*) exhibiting elevated levels of *AhSPL5* expression were chosen for a subsequent phenotypic characterization (Figure 10B).

Germination assays were employed to assess the salt tolerance of the *AhSPL5-OE* lines. The results indicate that the *AhSPL5-OE* lines exhibited higher rates of germination than the wild type when cultivated on 1/2 MS media with an addition of 100 mM NaCl (Figure 10A,C).

**Figure 11:**
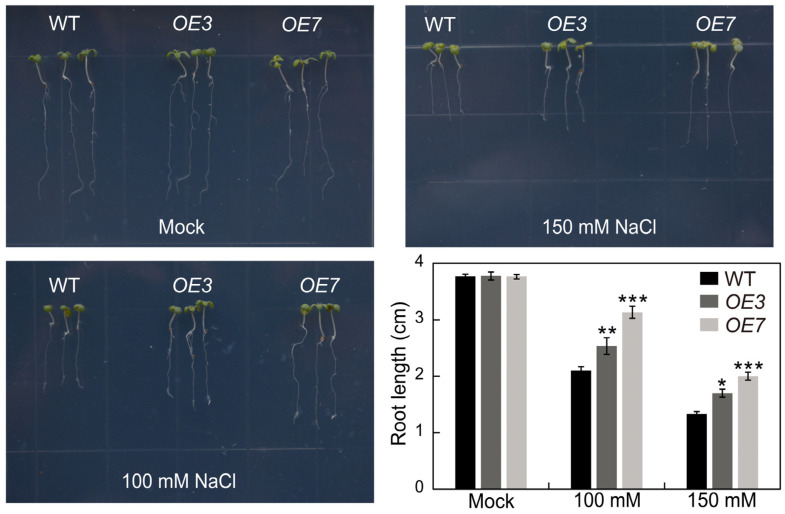
Evaluating the root development of the *AhSPL5-OE* lines and wild-type *Arabidopsis* when exposed to salt stress treatments of 100 and 150 mM NaCl. The length of the primary root was measured after seven days of growth. WT, wild-type. The data are the means ± SD from three independent replications. * *p* < 0.05, ** *p* < 0.01, *** *p* < 0.001 (*t*-tests).

Furthermore, a test was conducted to observe how the *AhSPL5-OE* lines reacted to salt stress by measuring root elongation. Following seven days of growth on 1/2 MS media supplemented with 100 and 150 mM NaCl, it was observed that the primary roots of the *AhSPL5-OE* lines exhibited a significantly greater length compared to those of the wild-type (WT) plants (Figure 11). Collectively, the findings from the salt treatment assays indicate that the overexpression of *AhSPL5* enhances salt stress tolerance in transgenic *Arabidopsis* plants.

### 2.10. AhSPL5 Enhances ROS-Scavenging Capability and Regulates the Expression of Stress-Responsive Genes

**Figure 12:**
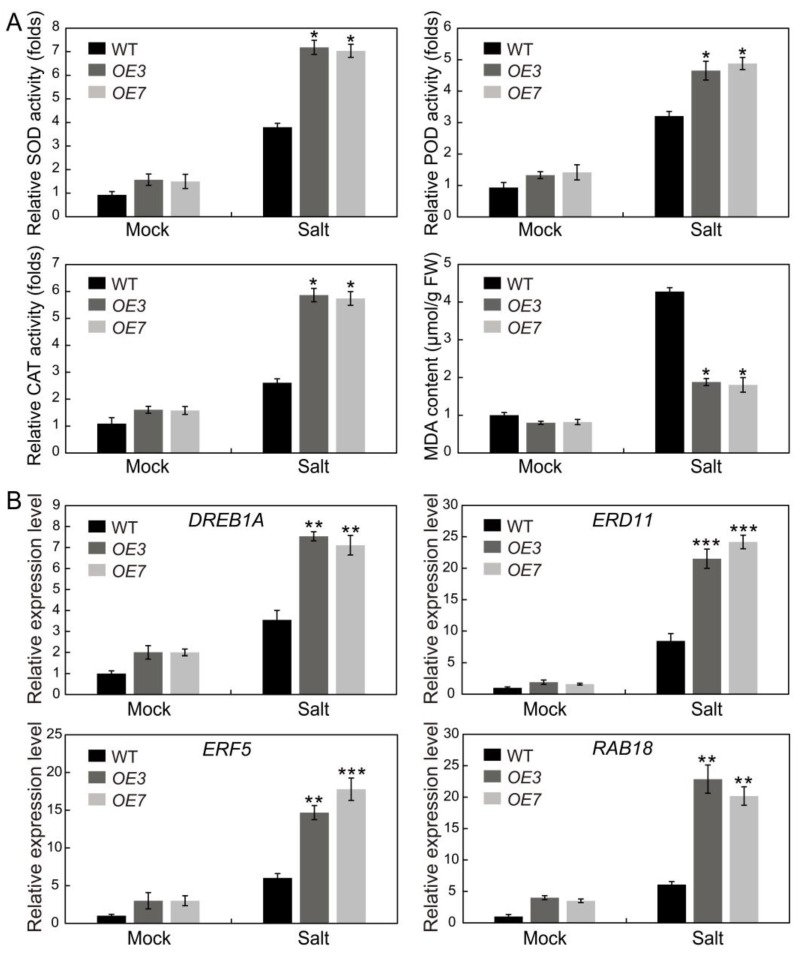
Variations in levels of antioxidant enzyme activity, MDA contents (**A**), and stress-responsive genes expression (**B**) in *AhSPL5-OE* lines and wild-type *Arabidopsis* following exposure to salt stress. MDA, malonic dialdehyde; CAT, catalase; POD, peroxidase; SOD; superoxide; WT, wild-type. The data are means ± SD from three independent replications. * *p* < 0.05, ** *p* < 0.01, *** *p* < 0.001 (*t*-tests).

Alterations in reactive oxygen species (ROS) levels are frequently linked to plant responses to abiotic stresses. To ascertain if variations in the antioxidant capacity are correlated with enhanced tolerance to salt stress in *AhSPL5-OE* plants, we assessed the stress-related physiological parameters of the *AhSPL5-OE* lines and wild-type (WT) plants with or without a salt stress treatment over a three-day period (Figure 12A). Under salt stress conditions, the activity of superoxide dismutase (SOD), peroxidase (POD), and catalase (CAT) in the *AhSPL5-OE* lines and wild-type (WT) plants were found to increase. Notably, the *AhSPL5-OE* plants exhibited a more pronounced enhancement of ROS-scavenging activities compared to the WT plants. Additionally, a significant decrease in dialdehyde (MDA) levels was observed in the *AhSPL5-OE* plants under salt stress conditions. The findings indicate that *AhSPL5* transgenic *Arabidopsis* plants may exhibit improved salt stress tolerance as a result of heightened ROS-scavenging capacity.

The function of AhSPL5 in controlling the salt response through the regulation of stress-responsive genes was examined using a qRT-PCR analysis. Under salt stress conditions, the stress-responsive genes were observed to be increased in expression in both the *AhSPL5-OE* lines and WT plants. Nevertheless, the *AhSPL5-OE* lines exhibited significantly higher expression levels of the stress-responsive genes compared to the WT plants (Figure 12B). These findings indicate that *AhSPL5* transgenic *Arabidopsis* might enhance salt tolerance by activating stress-responsive genes transcription.

## 3. Discussion

SPL proteins, a group of particular transcription factors found in plants, are crucial for plant development, growth, and adaptation to environmental stresses [23]. Furthermore, the *SPL* gene family has been identified in several plant species, such as the potato and tomato (fifteen members) [38,39], *Arabidopsis* and rice (seventeen and nineteen members, respectively) [10], and soybeans (forty-one members) [40]. However, the identification and analysis of the *SPL* gene family have not been reported on in an important oil crop, the peanut (*Arachis hypogaea* L.). The peanut is also susceptible to multiple abiotic stresses, such as drought and high salinity. Therefore, it is important to identify and analyze the peanut *SPL* genes in response to multiple abiotic stresses. In this study, a total of thirty-eight *AhSPL* genes were identified from the peanut genome and divided into seven groups, together with the *Arabidopsis SPL* genes (Figure 1). The number of *AhSPL* genes was overrepresented compared to that of *Arabidopsis*, which could be due to the fact that the peanut is an allotetraploid. All of the AhSPL members contained at least one zinc finger structure (Figure 3). Furthermore, the localization of all thirty-eight AhSPL proteins in the nucleus was predicted (Supplementary Table S1), with results comparable to those of the *Arabidopsis* SPL proteins [18]. Notably, a phylogenetic analysis, conserved motif, gene structure, chromosomal localization, duplication analysis, *cis*-acting elements analysis, and expression patterns analysis, as well as the prediction of the regulatory network and GO enrichment analysis, were performed for the peanut SPL family members.

The analysis of the conserved motifs and gene structure could help in understanding the evolution of the SPL family genes [41,42]. The analysis results show that the peanut SPL members within the same group have a similar motif arrangement and gene structure (Figure 2), supporting the reliability of the phylogenetic analysis and the evolutionarily conserved features of the peanut SPL family. Other plant species, such as the potato [38], tomato [39], and soybean [40], have been reported to exhibit a comparable gene structure and evolutionary relationship among the SPLs. A previous study showed that gene duplication events (segmental and tandem duplication) play a major role in gene family expansion [43]. In the present study, segmental duplication events were the main sources of the SPL family expansion in the peanut, rather than tandem duplication events (Figure 4B). Comparable results have also been obtained in other plant *SPL* gene groups, including maize [11], potato [38], and rice [10]. In addition, the twenty-four pairs of *AhSPL* genes all exhibited *Ka*/*Ks* values less than one (Supplementary Table S2), suggesting that purifying selection may have influenced the evolution of these *AhSPL* genes in the peanut. Furthermore, duplicated *AhSPL* genes were found to be present in the same evolutionary groups, such as *AhSPL4*/*AhSPL24* and *AhSPL8*/*AhSPL28* in group I, *AhSPL3*/*AhSPL21* and *AhSPL11*/*AhSPL32* in group II, and *AhSPL15*/*AhSPL35* in group III. However, duplication gene pairs for five *SPL* genes (*AhSPL13*, *AhSPL22*, *AhSPL23*, *AhSPL33*, and *AhSPL36*) were not found. This might be due to the loss during the evolution process of the *AhSPL* genes. Notably, two segmental duplication gene pairs (*AhSPL11*/*AhSPL32* and *AhSPL12*/*AhSPL31*) showed high expression levels in the pistil and veg.shoot of the peanut, respectively (Figure 6A).

In order to gain a deeper understanding of the potential biological functions of the *SPL* genes in peanut growth, development, and responses to biotic/abiotic stresses, a comprehensive analysis of the *cis*-acting regulatory elements of the *AhSPL* genes was conducted. This is helpful to better understand the expression of the *AhSPL* genes and their response to biotic/abiotic stresses. We found that the promoter regions of the *AhSPL* genes possess multiple regulatory elements (Figure 5). It has been reported that the abscisic acid response element (ABRE) may be involved in multiple abiotic stresses [44,45,46]. Notably, the presence of an ABRE element in the *AhSPL* gene promoters was partially associated with gene expression levels under abiotic stresses. For instance, the transcriptomic (RNA-seq) data indicate that *AhSPL16*, *AhSPL25*, and *AhSPL36* were up-regulated under drought and salt stresses (Figure 6B). Moreover, 86.8% (thirty-three of thirty-eight) of the *AhSPL* genes contain an unequal number of anaerobic stimulation elements (AREs). The AREs were first identified in the maize *Adh-1* gene promoter, and are induced by drought and cold stresses [47]. A total of 73.7% (twenty-eight of thirty-eight) of the *AhSPL* genes contain ethylene response elements (EREs), suggesting that these *AhSPL* genes may be involved in the peanut’s defense responses. Recent studies have shown that SPL transcription factors are capable of regulating immune responses in plants [48]. However, further experiments are needed to determine whether SPL proteins regulate peanut plant immunity. Furthermore, almost all the *AhSPL* genes contain development-related elements, such as the AAGAA motif. Taken together, the promoter analysis suggests that the *AhSPL* genes may functions in regulating peanut development and biotic/abiotic stresses.

The expression patterns in different tissues and under abiotic stresses can better reveal the potential biological functions of the *AhSPL* genes in the peanut. The results show that the *AhSPL* genes exhibited different tissue expression patterns (Figure 6A), implying the functional diversity of these genes during peanut growth and development. What is more, eighteen *AhSPL* genes were up-regulated under drought conditions, and five genes were up-regulated under salt conditions (Figure 6B). These findings align with prior studies that have shown an increased expression of multiple *SPL* genes in response to various stressors. For example, some *SPL* genes in alfalfa (*Medicago sativa* L.) are induced by drought, salt, and methyl jasmonate (Me JA) [49]. Several genes encoding SPL transcription factors demonstrated a notable increase in expression levels in *Fraxinus mandshurica* when exposed to ABA, cold, and salt treatments [50]. In addition, some *AhSPL* genes were also enriched for pathways acting in response to external stimulus (GO:0009605) (Figure 9). These results suggest the functional conservation of *SPL* genes in the regulation of environmental stresses.

Transcription factors are able to regulate gene expression at the transcriptional level. In *Arabidopsis*, the WRKY53 transcription factor can regulate leaf senescence progression by inhibiting the expression level of the *SENRK1* gene [51]. AhbHLH121 improves salt tolerance in the peanut by activating the expression of *AhPOD*, *AhCAT*, and *AhSOD* [52]. Significantly, it was observed that the *AhSPL* genes’ promoter regions contained multiple anticipated binding sites for transcription factors, such as ERF, NAC, MYB, Dof, and MICK_MADS (Figure 7). MicroRNAs (miRNAs) may also be implicated in *SPL*-regulated gene networks. Ten of the seventeen *SPL* genes discovered in *Arabidopsis* were identified as possible targets of miR156/157 [53]. Eighteen *SPL* genes in populus have been identified as potential targets of miR156 [54]. In this study, a total of twenty-seven identified *AhSPL* genes were potentially targeted by eleven miRNAs in the peanut (Figure 8). Among these, most of the *AhSPLs* are ahy-miR156 potential targets. Notably, salt and drought stress treatments can significantly induce the expression of *AhSPL5* (Figure 6B), suggesting that AhSPL5 might play a role in the response to salt and drought stresses. Further experiments indicated that the overexpression of *AhSPL5* can enhance salt tolerance in transgenic *Arabidopsis* (Figure 10 and Figure 11). Moreover, the overexpression of *AhSPL5* can enhance ROS-scavenging capability and promote the activation of stress-related genes (Figure 12), supporting the potential role of more *AhSPL* genes in the response to stresses.

## 4. Materials and Methods

### 4.1. Identification and Annotation of SPL Transcription Factor Family in Peanuts

The sequences of the sequenced peanut species were downloaded from the PeanutBase (https://legacy.peanutbase.org/peanut_genome, accessed on 22 September 2023). The SPL protein sequence data for *Arabidopsis thaliana* were obtained from the Arabidopsis Information Resource (TAIR, http://www.arabidopsis.org, accessed on 22 September 2023). First, the protein sequences of the *Arabidopsis* SPLs were utilized as queries in the BLASTP program against the peanut genome, employing an *E*-value threshold of 0.0001. Second, the HMM profiles of the SBP domain (PF03110) for SQUAMOSA-PROMOTER BINDING PROTEIN were employed to identify the peanut SPL protein sequences using HMMER (version 3.0), employing an *E*-value threshold of 0.0001. Each output peanut *SPL* gene was further examined using Pfam (http://pfam.xfam.org/search, accessed on 23 September 2023) [55] and SMART (http://smart.embl-heidelberg.de/, version 9, accessed on 23 September 2023) [56]. The theoretical isoelectric point (pI) and molecular weight (Mw) of the AhSPLs were examined using Expasy (http://web.expasy.org/protparam/, accessed on 24 September 2023) [57].

### 4.2. Multiple Sequence Alignment and Analysis of Phylogenetics

A multiple sequence alignment (MSA) of the SBP domain in the AhSPL proteins was performed using the DNAMAN tool (version 6, Lynnon Biosoft) [58]. A phylogenetic tree was created using MEGA software (version 6.06) [59] with 1000 bootstrap tests, based on the alignment result of the AhSPL and AtSPL protein sequences.

### 4.3. Gene Structure and Conserved Domains

The genetic composition of the AhSPL genes, encompassing coding sequences and introns, was examined through GSDS 2.0 software (http://gsds.cbi.pku.edu.cn/, accessed on 29 September 2023) [60]. The protein conserved domains were identified using the MEME program (version 5.5.3, https://meme-suite.org/, accessed on 29 September 2023) [61]. The maximum number of motifs was 10, with motif widths varying from 6 to 50 amino acids.

### 4.4. Chromosomal Location and Gene Duplication

The location data for the *AhSPL* genes were extracted from the peanut genome and TBtools (version 1.120) [62] was employed to perform the mapping of the *AhSPL* genes onto their respective chromosomes. The identification of tandem and segmental gene duplications was conducted using the MCScanX program [63], and the visualization of the obtained results was achieved through the utilization of Circos [64]. The syntenic analysis of orthologous genes between the peanut and four other plant species was conducted using TBtools (version 1.120) [62]. Subsequently, the rates of nonsynonymous (*Ka*) and synonymous (*Ks*) substitutions (*Ka*/*Ks*) were calculated using DnaSP (version 5.0) [65], considering the identification results of duplicated *AhSPL* genes.

### 4.5. Analysis of Cis-Acting Elements

The promoter sequences, located 2 kb upstream of the start codon (ATG), for thirty-eight *AhSPL* genes were obtained from the peanut genome. The PlantCARE (http://bioinformatics.psb.ugent.be/webtools/plantcare/html/, accessed on 25 September 2023) [66] database was utilized to identify potential *cis*-acting elements.

### 4.6. Expression Pattern Analysis

The RNA-seq data (accession number: PRJNA291488, SRR8177741, and SRP093341) were obtained from NCBI to analyze the *AhSPL* genes’ expression in 22 tissues and under salt and drought stress conditions [67,68,69]. The expression levels (FPKMs) of the *AhSPL* genes were transformed using log2. Subsequently, the expression patterns were visualized using TBtools (version 1.120) [62].

### 4.7. Prediction of Factors Involved in Regulating AhSPLs’ Expression

To explore the regulation of *AhSPL* gene expression by factors, the transcription factors (TFs) and miRNAs were predicted. The 2.0 kb promoter sequences of the *AhSPL* genes were submitted to the PlantRegMap website (http://plantregmap.gao-lab.org/, accessed on 26 September 2023) [70] to investigate the potential binding sites for the TFs. All the potential TFs were then visualized using TBtools (version 1.120) [62]. In addition, the coding region sequences of all the *AhSPL* genes were submitted to the psRNATarget server (http://plantgrn.noble.org/psRNATarget/, accessed on 26 September 2023) [71] for predicting the miRNAs. Cytoscape (version 3.9.1) [72] was used to generate the regulatory map.

### 4.8. GO (Gene Ontology) Enrichment Analysis

All the AhSPL protein sequences were submitted to the eggNOG website (http://eggnog-mapper.embl.de/, accessed on 27 September 2023) to perform the GO annotation analysis [73]. GO enrichment was then visualized using TBtools (version 1.120) [62].

### 4.9. RNA Extraction and qRT-PCR

RNA samples from *Arabidopsis* leaves were extracted with an Ultrapure RNA Kit from cwbiotech in Beijing, China, and then converted into cDNA using a PrimeScript™RT reagent Kit (TaKaRa). The determination of the expression levels of four stress-related genes from *Arabidopsis*, *DREB1A* (AB013815.1), *ERD11* (D17672.1), *ERF5* (NM_124094.3), and *RAB18* (X68042.1), was conducted. The *Arabidopsis Act2* gene served as the reference for the internal controls during the qRT-PCR reactions, which were carried out with 40 cycles on a Roche LightCycler 480 Real-Time PCR machine. Data from three replicates were obtained and analyzed using the 2^−ΔΔCT^ method. The primer sequences used in the current study are listed in Supplementary Table S4.

### 4.10. Generation of AhSPL5 Transgenic Arabidopsis Plants

To study the overexpression, the complete coding sequence (CDS) of *AhSPL5* was amplified with PCR, and then inserted to the pCHF3 vector at the *Sac* I site using Infusion (Clontech) technology, leading to the development of plasmid *35S::AhSPL5* after confirmation through sequencing. The constructed plasmid was then introduced into *Agrobacterium* GV3101 competent cells. Positive *Agrobacterium* colonies were identified and used to transform *Arabidopsis* Col-0 plants using the floral dip technique [74]. To produce *AhSPL5* overexpression plants, the T0 generation seeds were screened on 1/2 MS media containing 50 mg/L kanamycin. Following this, T3 lines with homozygous genotypes were chosen for additional phenotypic assessment.

### 4.11. Seedling Growth Assays

In the seed germination assays, *Arabidopsis* seeds were initially sterilized with 75% alcohol and subsequently distributed evenly onto 1/2 MS media supplemented with 100 mM NaCl. The seeds were subjected to stratification at 4 °C in darkness for 2 days before being transferred to a growth chamber set at 23 °C under continuous light conditions. Following a cultivation period of 6 days, the germination rates were determined with three replicates.

The *Arabidopsis* seedlings were first grown on 1/2 MS medium for a week prior to being moved to 1/2 MS medium with the addition of 100 mM and 150 mM NaCl. After an additional 7-day period of incubation, the primary root length was measured using three replicates.

### 4.12. Physiological Measurements

The physiological parameters were assessed for fully expanded leaves sourced from the plants subjected to salt-stressed conditions. The levels of malondialdehyde (MDA) and the enzymatic activities of superoxide dismutase (SOD), peroxidase (POD), and catalase (CAT) were quantified following established protocols [75], with three biological replicates conducted.

## 5. Conclusions

In this study, the peanut *SPL* gene family was investigated through the utilization of bioinformatics analysis, and a total of thirty-eight AhSPL members were identified. These members were classified into seven groups, along with their *Arabidopsis* homologs. The results offer an understanding of the different facets of the peanut *SPL* gene family, such as their physical and chemical characteristics, evolutionary connections, distribution of domains, location on chromosomes, composition of motifs, structure of genes, and patterns of expression. Moreover, a number of genes, including *AhSPL5*, *AhSPL16*, *AhSPL25*, and *AhSPL36*, showed increased expression levels in response to drought and salt conditions. The overexpression of *AhSPL5* is able to improve the ability of transgenic *Arabidopsis* to tolerate salt by enhancing its capacity to remove ROS and by promoting the activation of stress-related genes. The findings suggest that the *AhSPL* genes play a crucial role in controlling the peanut’s reactions to environmental stresses and growth. The precise function of each *AhSPL* gene in peanut growth and its reaction to stress needs to be confirmed in future research through the utilization of advanced genome editing and functional genomics techniques. This research establishes a theoretical basis for future studies on the roles of the *SPL* genes in enhancing the peanut’s resistance to abiotic stresses.

## Data Availability

All data supporting the findings of this study are available within the paper and within its Supplementary Materials published online.

## Supplementary Materials

The following supporting information can be downloaded at https://www.mdpi.com/article/10.3390/plants13081057/s1, Figure S1: The putative conserved motifs in AhSPL proteins; Table S1: Detailed information about the identified peanut SPL family members; Table S2: Detailed information about the segmental duplication gene pairs; Table S3: The syntenic pairs between the peanut and other four plant species; Table S4: The primers used in this study.
