# Supplementary figures and images for: A Comprehensive Analysis of the Peanut SQUAMOSA Promoter Binding Protein-like Gene Family and How AhSPL5 Enhances Salt Tolerance in Transgenic Arabidopsis
Source: Plants (Basel). 2024 Apr 9;13(8):1057. doi: 10.3390/plants13081057 (PMC11055087; doi:10.3390/plants13081057)

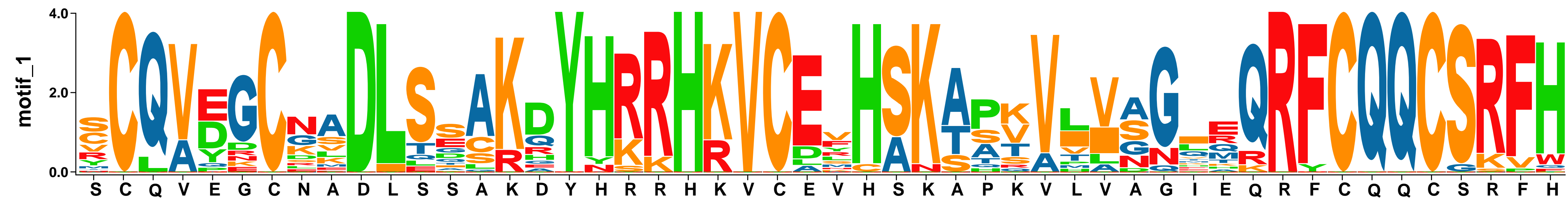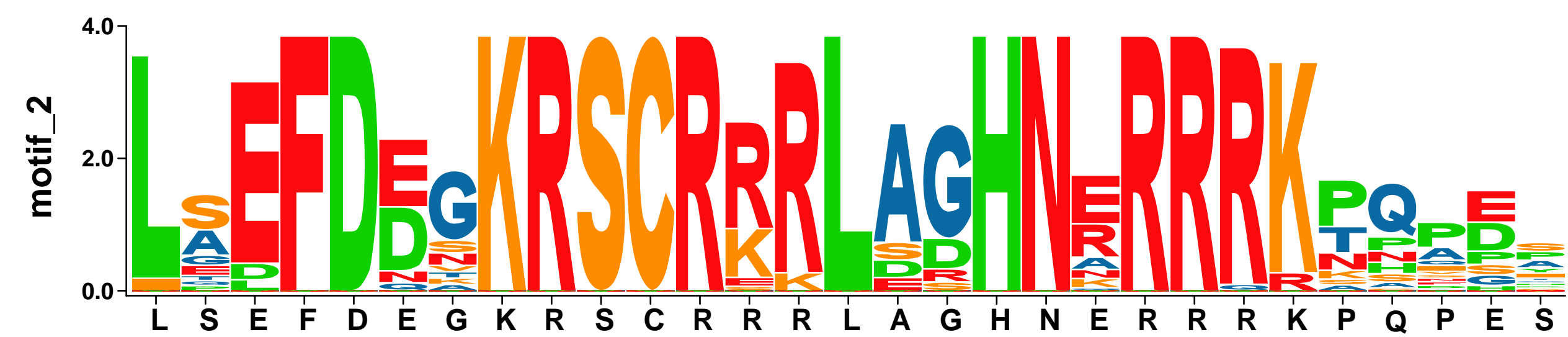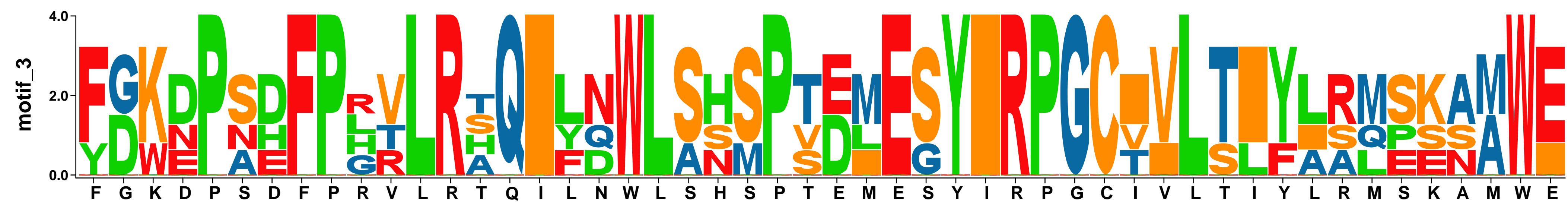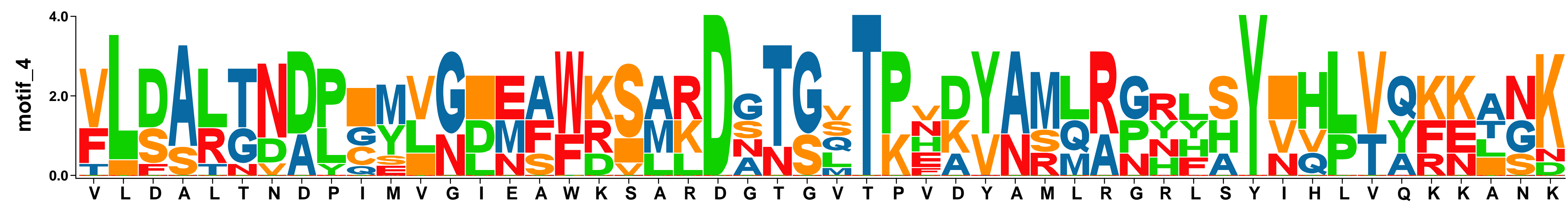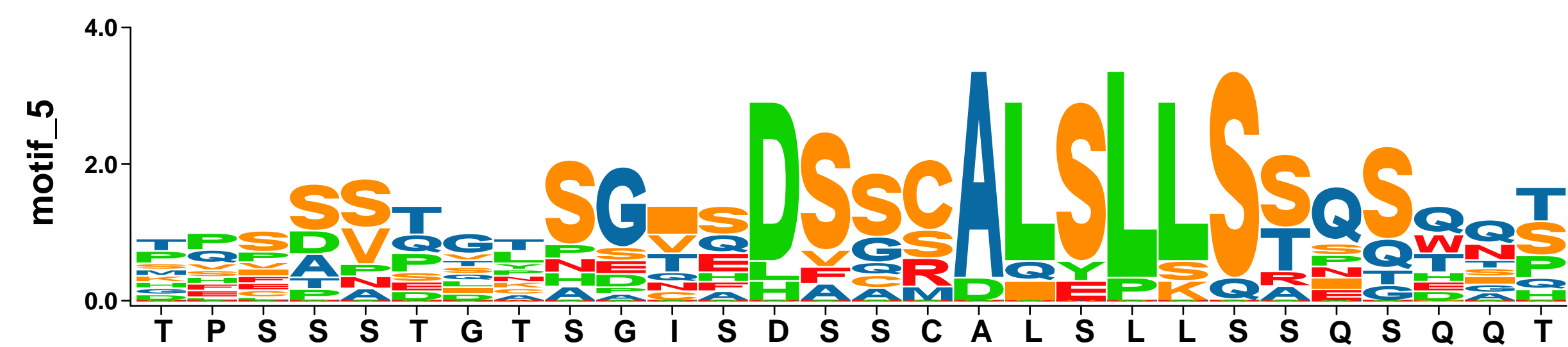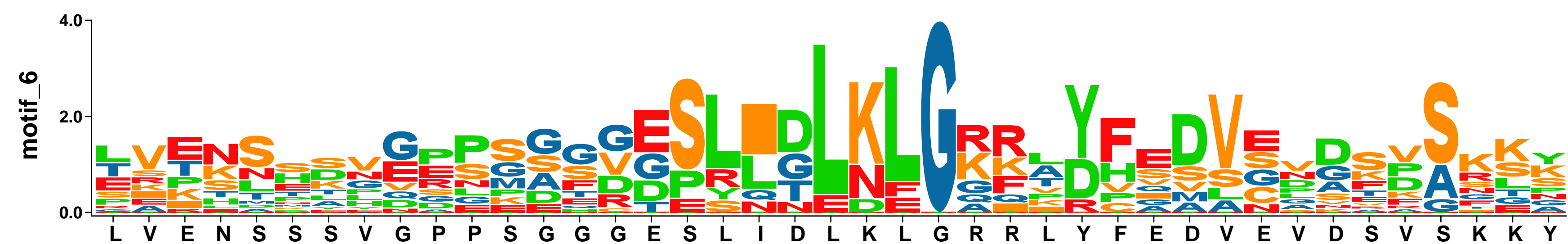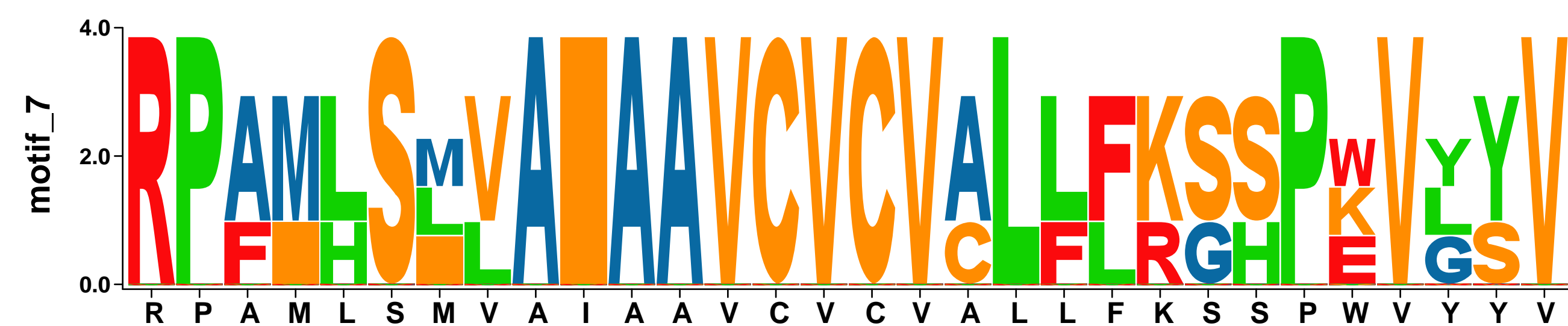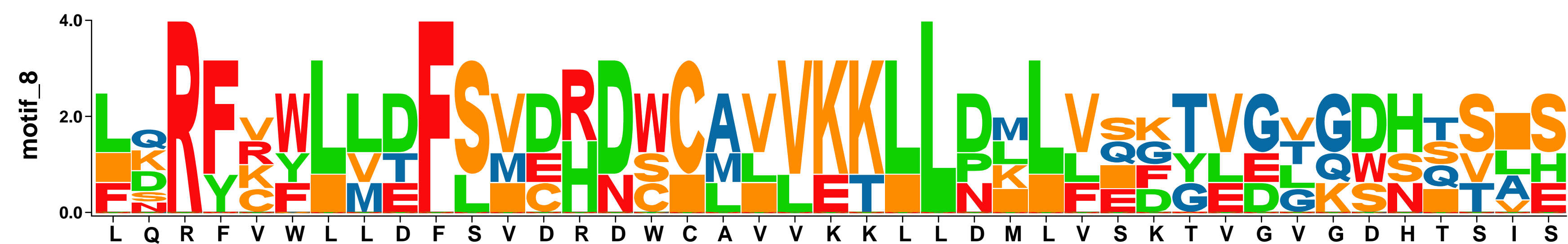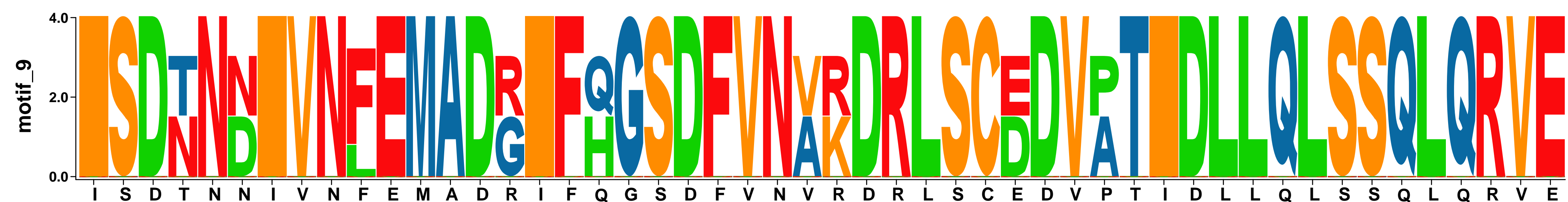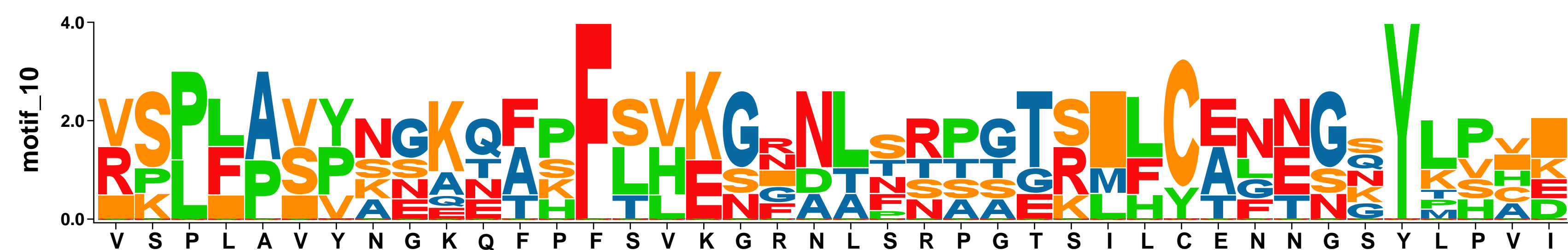

Supplement: Supplementary file 1 [file plants-13-01057-s001.zip › Supplementary Figure S1.pdf]
